# Interactions between Calcium and Alpha-Synuclein in Neurodegeneration

**DOI:** 10.3390/biom4030795

**Published:** 2014-08-14

**Authors:** Alex Rcom-H’cheo-Gauthier, Jacob Goodwin, Dean L. Pountney

**Affiliations:** Griffith Health Institute, School of Medical Science, Griffith University, Gold Coast, Queensland 4222, Australia; E-Mails: alexandre.rcom-hcheo-gauthier@griffithuni.edu.au (A.R.-H.-G.); j.goodwin@griffith.edu.au (J.G.)

**Keywords:** alpha-synuclein, Parkinson’s disease, calcium, multiple system atrophy, neurodegeneration, oxidative stress

## Abstract

In Parkinson’s disease and some atypical Parkinson’s syndromes, aggregation of the α-synuclein protein (α-syn) has been linked to neurodegeneration. Many triggers for pathological α-syn aggregation have been identified, including port-translational modifications, oxidative stress and raised metal ions, such as Ca^2+^. Recently, it has been found using cell culture models that transient increases of intracellular Ca^2+^ induce cytoplasmic α-syn aggregates. Ca^2+^-dependent α-syn aggregation could be blocked by the Ca^2+^ buffering agent, BAPTA-AM, or by the Ca^2+^ channel blocker, Trimethadione. Furthermore, a greater proportion of cells positive for aggregates occurred when both raised Ca^2+^ and oxidative stress were combined, indicating that Ca^2+^ and oxidative stress cooperatively promote α-syn aggregation. Current on-going work using a unilateral mouse lesion model of Parkinson’s disease shows a greater proportion of calbindin-positive neurons survive the lesion, with intracellular α-syn aggregates almost exclusively occurring in calbindin-negative neurons. These and other recent findings are reviewed in the context of neurodegenerative pathologies and suggest an association between raised Ca^2+^, α-syn aggregation and neurotoxicity.

## 1. Introduction

### 1.1. Neurodegeneration and α-Synuclein

Under normal cellular conditions, proteins have a stable fold that is appropriate to perform their biological function. In general, misfolded proteins that cannot be refolded are degraded by the ubiquitin proteasome system (UPS), or via lysosomal degradation. In disease, however, the misfolded protein may be resistant to degradation and undergo aggregate formation. Abnormal aggregation of the protein α-synuclein (α-syn) has been implicated in a number of neurological diseases, including Parkinson’s disease (PD), characterised by cytoplasmic aggregates in multiple cell types throughout the central nervous system. Collectively, these diseases are termed α-synucleinopathies and include PD and the atypical Parkinson’s syndrome, multiple system atrophy (MSA). In this report, key features of PD and MSA will be reviewed, two diseases that characterize the range of cell types affected by α-syn aggregates. Moreover, mechanisms of and factors influencing α-syn aggregation in the CNS will be discussed, with a special emphasis on the role of Ca^2+^ interactions [1,2,3].

### 1.2. Parkinson’s Disease and α-Synuclein

**Figure 1:**
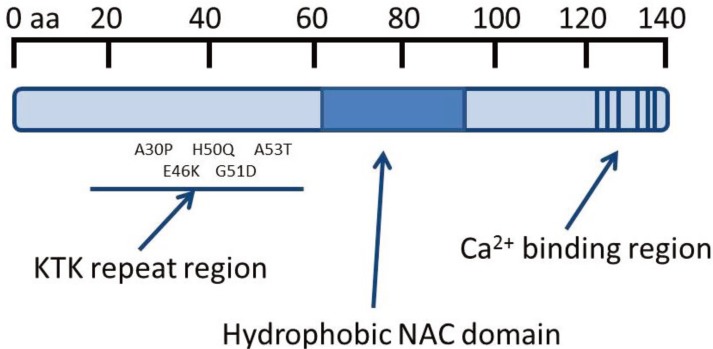
α-Synuclein protein (α-syn) domain structure. α-Syn contains three putative domains. KTK repeats in the *N*-terminus are involved in lipid interaction, the hydrophobic NAC domain is important for aggregation and the C-terminal Ca^2+^ binding site can increase the rate of oligomerization [1,2]. The Parkinson’s disease (PD)-linked point mutations are indicated within the neurotransmitter vesicle binding domain.

PD has progressive clinical symptoms, including slight weakness, tremors, forward posture, sleep disturbance, constipation, the inability to walk unaided, speech impairment, difficulty swallowing, tremor, loss of urinary and gastrointestinal control and extreme exhaustion. The three main symptoms of PD are bradykinesia, or a slowing of voluntary controlled movement, rigidity, and tremor. Pathological examination of brain tissues from PD sufferers shows that there is a loss of dopaminergic neurons in the Substantia nigra (SN), a region of the brain that, through neural connections with the striatum, is responsible for controlled muscle movements. PD can be classified into two main groups: idiopathic and familial. Idiopathic PD, the larger of the two groups, accounts for roughly 85%–90% of all PD cases. Familial PD accounts for the remaining 10%–15% of cases and is due to mutations in a number of genes, including *SNCA* (*PARK1/4*), the gene responsible for the expression of α-syn. To date, five PD-linked point mutations in *SNCA*, have been identified [1], comprising the A30P [4], A53T [5], E46K [6], G51D [7] and H50Q [8] amino acid substitutions that disrupt the neurotransmitter vesicle binding domain (see Figure 1). The A53T and A30P mutations also affect the response to oxidative stress with expression of these mutant isoforms significantly increasing cytotoxicity induced by hydrogen peroxide and 1-methyl-4-phenylpyridinium (MPP^+^) in comparison to cells expressing wild-type α-syn and control cells [9]. Moreover, the A30P, A53T and H50Q mutations result in increased oligomerization and fibril formation compared to wild-type [10,11]. Furthermore, gene duplication [12] and triplication of α-syn [13] have also been found in familial PD, implicating gene dosage effects in pathogenesis.

### 1.3. Parkinson’s Disease and Environmental Factors

Environmental factors, such as exposure to pesticides and insecticides, may play a role in the pathogenesis of idiopathic PD. Rotenone, 1-methyl-4-phenylpyridinium (MPP+) and Paraquat have all been linked to PD [14,15]. These chemicals generate oxidative stress through the inhibition of complex I of the mitochondrial electron transport chain (rotenone, MPP+) or by acting as a general inducer of reactive oxygen species (ROS) (paraquat). Interestingly, two environmental factors have the opposite affect and are protective for PD. Cigarette smoking and caffeine have both been shown to be protective [16], and while the mechanism of caffeine protection is unclear the caloric restriction associated with smoking with a subsequent decrease in metabolic oxidative stress may be a factor.

### 1.4. Pathology of Parkinson’s Disease and Multiple System Atrophy

A characteristic pathological feature of PD is a severe loss of the neuromelanin-positive dopaminergic neurons of the SN, located in the midbrain. Signalling between the SN and the striatum is involved in controlling muscle movements; therefore the resultant loss of nigro-striatal pathway signalling can explain the classical symptoms of PD. Of the neurons that remain, large protein aggregates called Lewy bodies (LB) are often observed. While LB’s are made up of numerous proteins, including proteasome components, lysosome components and chaperone proteins, α-syn immunoreactivity defined α-syn as a major component of the LB in PD and in dementia with Lewy bodies [1,17].

Multiple system atrophy (MSA) is also late onset, neurodegenerative, idiopathic and progressive [3]. However unlike PD, where aggregates form in neuronal cells, α-syn positive protein inclusions are formed within the cytoplasm of oligodendrocytes and are therefore termed glial cytoplasmic inclusions (GCI). MSA also differs from PD in the distribution of these cytoplasmic aggregates throughout the central nervous system where in addition to the SN, the locus coeruleus, putamen, inferior olives, pontine nuclei, Purkinje cells and the intermediolateral columns of the spinal cord are also affected. Classified into two subgroups, MSA-P (Parkinsonian) is characterized by GCIs within the SN, which results in the Parkinsonian symptoms, whereas, MSCA-C (cerebellar ataxia) affects the cerebellum and results in gait and limb ataxia and oculomotor disturbances. In each sub-type, the severity and type of symptoms is dependent on the distribution and density of α-syn inclusions. MSA has no strong genetic link, although a number of single nucleotide polymorphisms, including the *SNCA* gene, have been identified with an increased risk of MSA [18]. A number of studies have looked at environmental and other risk factors for MSA, although with no consensus findings [19,20].

### 1.5. Properties of α-Synuclein

α-Syn is a 14 kDa protein encoded by the *SNCA* gene that is highly conserved in vertebrate species. Although the exact role of α-syn remains unclear, the protein is primarily expressed in the olfactory bulb, frontal cortex, striatum and the hippocampus with lower expression levels also observed in the hypothalamus, thalamus, midbrain, cerebellum and pons [21] where it localises to presynaptic terminals of dopaminergic neurons [22,23]. It is believed to be involved in neurotransmitter vesicle recycling and dopamine neurotransmission through interaction with soluble NSF attachment protein receptor (SNARE) [24]. Three putative domains of the α-syn protein have been identified (see Figure 1). The N-terminal domain comprises seven 11 amino acid imperfect repeat sequences, predicted to form aliphatic helices (KTK repeats) allowing α-syn to associate with lipid membranes. The C-terminal domain contains a number of acidic residues identified as a Ca^2+^ binding site [25]. The third domain (NAC domain) contains hydrophobic amino acids and is important for aggregation [26]. The conformation of α-syn is highly dependent on environmental conditions. In the aqueous cellular environment, α-syn adopts a random coiled structure, but adopts a helical conformation upon binding to acidic phospholipid vesicles [27,28,29]. α-Syn is also prone to nucleation dependent aggregation [30] through the N-terminus [31] and this aggregation, inhibited upon membrane binding, transforms α-syn from the random coiled conformation to beta-pleated sheets [32,33]. Deletion of the hydrophobic 12 aa central region of α-syn results in the loss of α-syn aggregation and the hydrophobic 12 aa region alone is sufficient to form aggregates. The α-syn protein has been shown to interact with membranes [34] and both the N- and C-terminus of the protein can be bound to membranes. This membrane interaction, and the function as a SNARE associated protein is mediated by Rab3a [35].

Gene triplication and duplication of *SNCA* in autosomal dominant forms of PD suggests the importance of α-syn gene dosage and protein concentration in aggregation. Interestingly, mouse studies have shown that there is an age-dependent decline in both α-syn protein and mRNA levels [36]. Whereas, analysis of single dopaminergic neurons from tissue of PD affected individuals and controls have revealed that there is a significant increase in α-syn mRNA levels [37].

### 1.6. α-Synuclein Oligomerization and Cytotoxicity

Abnormal oligomeric α-syn species have been implicated in the pathogenesis of α-synucleinopathies [38,39,40,41,42,43]. Danzer and co-workers found that small annular α-syn species but not monomeric protein were able to increase cytosolic Ca^2+^ levels in SH-SY5Y cells [43]. This increase of cytosolic Ca^2+^ was rapid, reaching a plateau around 200 s, and dependent on extracellular Ca^2+^, indicating a pore forming ability of α-syn oligomeric species. Treatment with α-syn oligomers resulted in an increased level of cleaved (active) caspase 3 indicating α-syn olimomers induced apoptosis. Outeiro *et al.* [38] used protein fragment complementation assay (PCA) and bimolecular fluorescence complementation (BiFC) assay to monitor α-syn monomer interaction *in vitro* and determined that the most favourable α-syn interaction was anti-parallel, resulting in cytotoxicity that could be suppressed by HSP70.

One possible mechanism for increased rate of α-syn oligomerization is an increase in mitochondrial mediated ROS production. Using PD cybrids, which have relatively high levels of oxidative stress and an enhancement in oligomer formation, treatment with CoQ10 and GSH antioxidants resulted in a decrease in oligomer formation [39]. Moreover, α-syn oligomer association with mitochondria may be linked to mitochondrial dysfunction [42]. Recently, using both non-denaturing gel electrophoresis and MALDI-TOF mass spectroscopy, α-syn has been shown to exist as α-syn tetramers in M17D, HEK293, HeLa and COS-7 cells [44,45], with predominantly helical structure. These normally occurring tetramers are proposed to be non-toxic, with the abnormal, toxic oligomeric species produced via interconversion first to the monomer.

### 1.7. α-Synuclein Post-Translational Modifications

Phosphorylation of α-syn has been shown to be important in increasing the rate of aggregation [46] and has also been shown to aid metal ion association [47,48]. Truncation and proteolytic processing of α-syn have also been implicated in aggregate pathology [49]. Oxidation leads to other common post-translational modifications, including nitrosylation, and it has been shown that oxidative stress can stabilise oligomeric α-syn species via the formation of di-tyrosine cross links [50,51]. However, oxidation of recombinant human α-syn results primarily in oxidation of methionine residues [52] and at normal physiological pH this oxidation abolishes α-syn aggregation [53]. One of the most striking characteristics of PD is the selective loss of dopaminergic neurons in the SN which has been mimicked in a drosophila model system [54]. However, α-syn has been shown to play a cyto-protective role in dopaminergic cells. The N27 dopaminergic cell line transfected with human α-syn is protected against MPP+ induced apoptosis via inhibition of PKCδ cleavage and inhibition of BAD, and the concentration of ROS is reduced, in comparison to non-transfected cells [55]. Dopamine has also been shown to inhibit the formation of α-syn fibrils via oxidative modification of the protein and enhances formation of protofibrillar α-syn species [56].

### 1.8. Exosomes and the Cell to Cell Spread of α-Synuclein

One factor contributing to the toxicity of oligomeric α-syn species is cell to cell spread of α-syn via exosomes [57]. Exosomes are membranous vesicles released from mammalian cells and have been shown to contain mRNA, microRNA and proteins. Alvarez-Erviti *et al.* [58] demonstrated that α-syn positive exosomes isolated from a SHSY-5Y neuroblastoma α-syn overexpressing cell line were capable of transferring the protein to other SHSY-5Y cells. They also concluded that inhibition of the lysosomes that are involved in α-syn degradation resulted in an increase in α-syn exosome-mediated release into the culture media. Danzer *et al.* [59] demonstrated in H4 cells and primary cortical neurons that α-syn in exosomes was oligomeric. They also deduced that α-syn was predominantly either outside or associated with the outer membrane of the exosome, but not totally excluded from the lumen. Furthermore, processing of disease-associated α-syn in the human brain is consistent with prion-like cell-to-cell spread [60]. Microglial cells have also been shown to secrete α-syn positive exosomes [61]. Moreover, it was found in foetal tissue graft recipients that return of symptoms of a long term graft survivor correlated with classic PD markers such as α-syn and ubiquitin aggregation within the grafted region, replicated in patients who received foetal mesencephalic dopaminergic neurons [62,63].

### 1.9. Oxidative Stress

There is evidence that oxidative stress is increased in normal aged brain however the level of oxidative stress is greatly increased in patients with neurodegenerative diseases [64]. The major contribution to oxidative stress in ageing primates originates from mitochondrial complexes I and III of the electron transport chain leading to greater mitochondrial DNA damage compared to nuclear DNA damage [65]. Quilty *et al.* [66] showed that when mouse primary neocortical cells were incubated in the absence of antioxidants a subset of neurons exhibited a higher α-syn expression and decreased apoptosis. Whereas, Selkoe and co-workers found that prefibrillar α-syn promoted complex I-dependent mitochondrial dysfunction [43]. Moreover, in human SHSY-5Y cells, there was no significant difference in the viability of cells with WT α-syn overexpression; however the A53T and A30P mutations were more susceptible to oxidative insult [9].

## 2. Increased Intracellular Ca^2+^ Induces α-Synuclein Oligomers

### 2.1. The Role of Ca^2+^ in the Neuron and Age Related Changes

Ca^2+^ plays many important roles in normal cellular processes, such as apoptosis, metabolism, signal transduction, gene expression and cell death, and intracellular Ca^2+^ homeostasis is tightly regulated between the cytoplasm, intracellular Ca^2+^ stores, such as the endoplamic reticulum (ER), and between the intracellular environment and the extracellular environment. Resting intracellular Ca^2+^ is found not to be increased with age of the neuron, with studies indicating no difference between intracellular Ca^2+^ levels of young and aged neurons. However, the return time to resting levels after a stimulus is greatly reduced in aged neurons. Thus, the possible link between Ca^2+^ and α-syn aggregation in neurodegenerative diseases is a current focus of research [67,68]. Indeed, α-syn oligomers have been shown to promote Ca^2+^ influx [42]. Furthermore Ca^2+^ and Co^2+^ binding have been shown to accelerate the formation of α-syn annular oligomeric species [69]. Although the precise Ca^2+^ binding site has not yet been defined, truncation at residue 125 was found to abolish Ca^2+^ binding and Ca^2+^-dependent aggregation.

The major sources of intracellular Ca^2+^ include Ca^2+^ influx through ligand-gated glutamate receptors, such as *N*-methyl-d-aspartate receptor (NMDAR) or various voltage-dependent Ca^2+^ channels (VDCCs), as well as the release of Ca^2+^ from intracellular stores. Clearance of Ca^2+^ after stimulation is achieved either by intracellular Ca^2+^ binding, uptake into the ER and mitochondria or pumping into the extracellular space via plasma membrane Ca^2+^ ATPases, which have been shown to be impaired in aged neurons [70,71,72]. Aged neurons also exhibit a decreased capacity to recover from Ca^2+^ stimulus through uptake into the intracellular Ca^2+^ stores with a decline in sarcoplasmic ER Ca^2+^ ATPase Ca^2+^ function [73]. The mitochondrion is a second intracellular Ca^2+^ store and like the ER, the ability of this organelle to act as a reservoir for Ca^2+^ is also decreased with age [74].

Calbindin (CB), calretinin and parvalbumin are three cytosolic calcium binding proteins that are capable of Ca^2+^ buffering in neurons. Bu *et al.* [75] found a decrease in both calretinin and CB in aged compared to young cortical neurons; put no difference in parvalbumin positive neurons. German *et al.* [76] found that in both idiopathic PD and in MPTP monkey or mouse models that CB+ neurons were spared. Moreover, calretinin expression in dopaminergic neurons of the SN protected against 6-hydroxydopamine [77,78]. Furthermore, Yamada *et al.* [79] found relative sparing of SN neurons positive for CB in PD cases.

### 2.2. Increased Intracellular Ca^2+^ Induces α-Synuclein Oligomers

**Figure 2:**
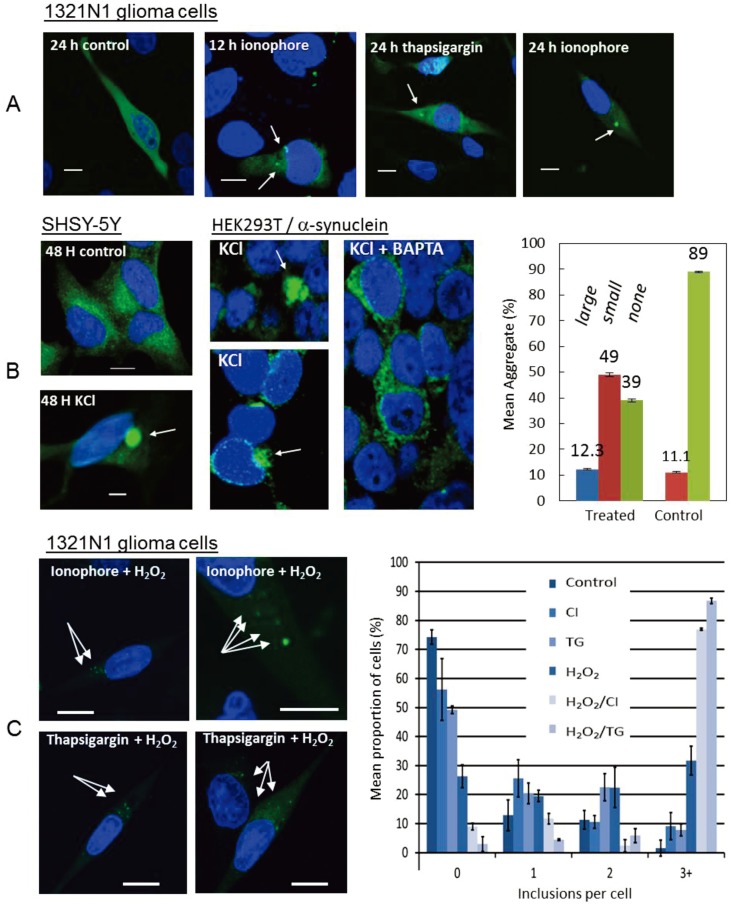
Raised intracellular Ca^2+^ promotes α-syn aggregation. (**A**) 1321N1 human glioma cells treated with either thapsigargin or Ca^2+^ ionophore caused raised intracellular free Ca^2+^ and induced α-syn aggregates (arrows) after 12–24 h [80]; (**B**) Potassium depolarization of SH-SY5Y human neuroblastoma and HEK293T cells resulted in transiently raised intracellular free Ca^2+^ and Lewy body-like large α-syn aggregates (arrows) that could be blocked by the BAPTA-AM Ca^2+^ chelator [81]. (**C**) Co-treatment of 1321N1 cells with thapsigargin (TG) or Ca^2+^ ionophore (CI) and hydrogen peroxide resulted in increased α-syn aggregates (arrows; graph, right); consistent with a cooperative interaction between raised free Ca^2+^ and oxidative stress [87]. Scale bars, 10 μm.

Recent studies have shown that a transient increase in the intracellular free Ca^2+^ concentration induced in cultured 1321N1 glioma cells by thapsigargin or Ca^2+^ ionophore (CI) chemical treatments caused a significant increase in the proportion of cells bearing microscopically-visible α-syn aggregates (Figure 2A). It was also demonstrated that chelating free Ca^2+^ with BAPTA, resulted in no significant difference in the number of inclusions between control and CI/BAPTA cells, indicating that raised intracellular free Ca^2+^ directly induces α-syn aggregates. Moreover, supporting studies with recombinant protein indicated that direct binding of Ca^2+^ ion to α-syn promoted rapid oligomer formation *in vitro* [80], which was not observed with the C-terminally truncated protein (1–125) that lacks the glutamate-rich putative Ca^2+^ binding domain [69] (see Figure 1). Further studies are needed to map the Ca^2+^ binding site by mutating each of the putative glutamate residues in this region thought to represent potential metal ligands (see Figure 1). More recently, Follett *et al.* [81], demonstrated that potassium depolarization of the plasma membrane in HEK293T and SH-SY5Y human cell lines resulted in raised intracellular free Ca^2+^ and α-syn aggregate formation under more physiologically relevant cellular conditions ([81]; Figure 2B). Both raised free Ca^2+^ and α-syn aggregation were blocked by BAPTA chelation treatment (Figure 2B, centre). Potassium depolarization was observed especially to trigger formation of frequent large, Lewy body-like perinuclear α-syn inclusion bodies (Figure 2B, right).

**Figure 3:**
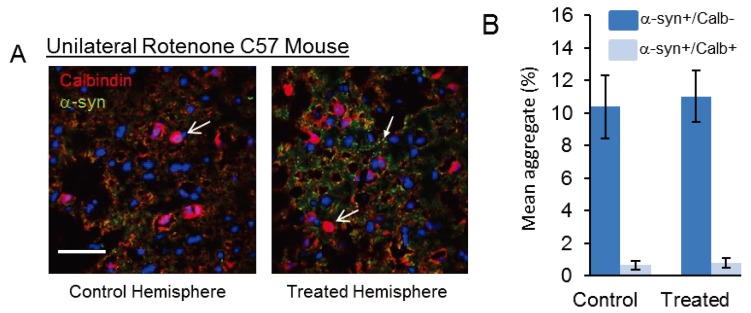
Unilateral rotenone lesion mouse (oxidative stress) model of PD shows α-syn aggregates primarily in calbindin-negative neurons. (**A**) CB+ neurons (arrowheads) showed relative protection in the unilateral rotenone lesion (oxidative stress) model of PD (as detailed in [85]), with more CB+ neurons surviving in the treated than in the untreated hemisphere and partitioning of α-syn aggregates (arrow) in the CB− neurons [86]. Scale bar, 50 μm. (**B**) Graph of cell counting data shows a significantly greater number of α-syn aggregates occur in CB− neurons than in CB+ neurons.

It is clear that raised Ca^2+^ is an important factor influencing α-syn aggregation, and potentially in the formation of the cytotoxic oligomeric species seen in disease. Thus, addition of Ca^2+^ to α-syn monomer *in vitro* could promote α-syn oligomerization and resulted in the rapid formation of potentially toxic annular α-syn oligomeric structures [80]. This provides a potential therapeutic target, by using drugs that modulate the amount of free Ca^2+^ in the cell. Ca^2+^ channel blockers, such as those from the dihydropyridine family, may be used to lessen the increase in intracellular Ca^2+^ seen in aged neurons [82]. A step towards replicating the complex architecture of the CNS and assessing Ca^2+^ blockade was performed by Chan *et al.* [83] who used brain slices prepared from a MPTP mouse model for PD. They found that by using blocking L-type Ca_v_1.3 Ca^2+^ channels with Isradipine, a common drug used to treat high blood pressure, they could recover dopaminergic neural activity. This supports the data of Yamada *et al.* [79] that dopaminergic neurons of the SN, rich in the Ca^2+^ binding protein CB, were preferentially spared in control brain sections compared with PD patients and the PD mouse model data showing neurons expressing CB were spared from pathological loss [84]. Consistent with this, Trimethadione (TMO), a Ca^2+^ channel blocker with broad selectivity commonly used as an anti-epileptic drug, blocked K^+^-depolarization induced Ca^2+^ influx into SH-SY5Y cells resulting in loss of α-syn positive aggregate formation post-depolarization [81]. Furthermore, recent studies using a unilateral rotenone (oxidative stress) lesion mouse model of PD (described in [85]), also showed improved survival of CB+ neurons and almost exclusive partitioning of α-syn aggregates in the CB− cell population (Figure 3; [86]). In this model, injection of rotenone into the medial forebrain bundle of one brain hemisphere only, allows for comparison of α-syn inclusion body positive and CB+ neurons between treated and untreated hemispheres.

### 2.3. α-Synuclein Oligomerization Induces Raised Ca^2+^ and Oxidative Stress

Elevated levels of intracellular α-syn have been shown to elevate levels of intracellular Ca^2+^ [88]. Secreted α-syn induces increase in capacitive Ca^2+^ entry in differentiated SH-SY5Y [89]. Reznichenko *et al.* [90] investigated Ca^2+^ dynamics in transgenic (tg) mice expressing human WT α-syn. α-Syn-tg mice exhibited augmented, long-lasting Ca^2+^ transients characterized by considerable deviation from the exponential decay. Furthermore control and α-syn KO groups demonstrated low percentages of neurons with Ca^2+^ abnormalities whereas the α-syn tg group showed Ca^2+^ response alteration, suggesting these alterations are related to α-syn expression.

Other studies have shown that α-syn overexpression augments mitochondrial Ca^2+^ transients by enhancing ER-mitochondria interactions. Cali *et al.* [91] demonstrated that α-syn with acidic C-terminal domain overexpression increases mitochondrial Ca^2+^ in SH-SY5Y and HeLa cells. Additionally, treatment with naturally secreted α-syn increases Ca^2+^ entry in primary rat cortical neurons and induces mitochondrial Ca^2+^ uptake. Significantly higher levels of mitochondrial Ca^2+^ content in α-syn treated cells were observed compared to control cells.

Dryanovsky *et al.* [92] found that in CB− and CB+ dopaminergic neurons having inclusions, mitochondrial oxidant stress levels were higher in the soma and proximal dendrites than in neurons without inclusions. Treatment with isradipine significantly diminished the oxidative stress levels in CB− dopaminergic neurons. This suggests that the formation of α-syn inclusions stimulates ROS production in the cytosol. α-Syn provokes an elevation of cytosolic Ca^2+^ in yeast that coincides with an increase in oxidative stress [93], suggesting that α-syn aggregation leads to an increase in mitochondrial Ca^2+^ transient and then to oxidative stress. WT α-syn has also been shown to induce mitochondrial NO when it is associated with mitochondria [94]. This indicates that not only will normal increases in oxidative stress cause aggregation but that aggregation of α-syn also induces more oxidative stress within the cell forming a positive feedback loop. However, this is contrary to other research which shows that α-syn protects cells from oxidative stress by inactivating the c-Jun *N*-terminal kinase (JNK) pathway [95].

### 2.4. Synergistic Effect of Ca^2+^ and Oxidative Stress

Oxidative stress is strongly implicated in α-synucleinopathy and may combine synergistically with other factors, such as α-syn expression and raised Ca^2+^, to promote α-syn aggregation and neurodegeneration. The combination of oxidative stress and α-syn expression has been used to generate a model of MSA in mice, whereby the overexpression of α-syn in glial cells is combined with 3-nitropropionic acid [96]. Moreover, recent studies have examined the role of oxidative stress in the formation of potentially toxic α-syn oligomeric species in conjunction with Ca^2+^ binding. It was found that, when treated with Ca^2+^ ionophore (CI) or thapsigargin (TG) and H_2_O_2_ in combination, there was a dramatic increase in the number of protein aggregates per cell in 1321N1 cells (Figure 2). This was also reflected in *in vitro* experiments that showed that the combination of Ca^2+^ treatment and oxidation of recombinant α-syn monomer caused the formation of stable, oligomeric α-syn aggregates, indicating a cooperative interaction between Ca^2+^ binding to α-syn and α-syn oxidation [87]. Thus, these recent findings have indicated that increased intracellular free Ca^2+^ and oxidative stress work synergistically to induce α-syn aggregation. This may be extremely important in the pathogenic mechanisms behind α-syn aggregation and α-synopthathy disease progression as fibrillization of α-syn is highly dependent on nucleation centres, with pre-aggregated α-syn dramatically increasing the rate of α-syn aggregation. Furthermore, kinetic studies of α-syn aggregation by Nath *et al.* [97] were consistent with an auto-catalytic mechanism. Thus, Ca^2+^/oxidation stabilized α-syn aggregates may serve to increase nucleation centres in disease. Indeed, the formation of di-tyrosine cross linked α-syn dimers have been found previously to be a rate-limiting step in the fibrillation process and the formation of nucleation centres [98]. Moreover, Ca^2+^ influx into neurons can induce oxidative stress in mitochondria of mouse dopaminergic neurons [99], as oxidative stress induced by Ca^2+^ influx was exacerbated in DJ-1 mutant mice.

## 3. Conclusions: Targeting Calcium with Future Therapeutics

The link between PD and some atypical Parkinson’s syndromes and aggregation of α-syn makes this process a major target for the development of future neurodegenerative therapies. Many triggers for pathological α-syn aggregation have been identified, including raised Ca^2+^ and oxidative stress. Recent studies have found that transient increases of intracellular Ca^2+^ induce cytoplasmic α-syn aggregates, that can be blocked by Ca^2+^ buffering or Ca^2+^ channel blocking agents. Furthermore, it has been shown that Ca^2+^ and oxidative stress cooperatively promote α-syn aggregation. These recent findings suggest an association between raised intracellular Ca^2+^, α-syn aggregation and neurotoxicity paving the way for the development of therapeutics that target raised Ca^2+^ [100]. Since Ca^2+^ lowering medications, such as anti-epileptic drugs, often have significant side-effects, successfully targeting raised intracellular free Ca^2+^ in the brain as a neuroprotective strategy will depend on the development of reliable genetic, imaging or biochemical tests. It is clear that in order to differentiate disease sub-types with strong Ca^2+^ involvement, multiple marker evaluation will be necessary.
